# *Melissa officinalis* L. Aqueous Extract Exerts Antioxidant and Antiangiogenic Effects and Improves Physiological Skin Parameters

**DOI:** 10.3390/molecules26082369

**Published:** 2021-04-19

**Authors:** Simona Sipos, Elena-Alina Moacă, Ioana Zinuca Pavel, Ştefana Avram, Octavian Marius Crețu, Dorina Coricovac, Roxana-Marcela Racoviceanu, Roxana Ghiulai, Ramona Daniela Pană, Codruţa Marinela Şoica, Florin Borcan, Cristina Adriana Dehelean, Zorin Crăiniceanu

**Affiliations:** 1 Department of Biochemistry and Pharmacology, Faculty of Medicine, “Victor Babeș” University of Medicine and Pharmacy, Eftimie Murgu Sq. no. 2, 300041 Timișoara, Romania; sipos.simona@umft.ro; 2 Department of Toxicology and Drug Industry, Faculty of Pharmacy, “Victor Babeș” University of Medicine and Pharmacy, Eftimie Murgu Sq. no. 2, 300041 Timișoara, Romania; alina.moaca@umft.ro (E.-A.M.); dorinacoricovac@umft.ro (D.C.); cadehelean@umft.ro (C.A.D.); 3 Research Centre for Pharmaco-Toxicological Evaluation, “Victor Babeș” University of Medicine and Pharmacy, Eftimie Murgu Sq. no. 2, 300041 Timișoara, Romania; babuta.roxana@umft.ro (R.-M.R.); roxana.ghiulai@umft.ro (R.G.); codrutasoica@umft.ro (C.M.Ş.); 4 Department of Pharmacognosy, Faculty of Pharmacy, “Victor Babeș” University of Medicine and Pharmacy, Eftimie Murgu Sq. no. 2, 300041 Timișoara, Romania; 5 Department of Surgery, Faculty of Medicine, “Victor Babeș” University of Medicine and Pharmacy, Eftimie Murgu Sq. no. 2, 300041 Timișoara, Romania; tavicretu@yahoo.com; 6 Department of Pharmaceutical Chemistry, Faculty of Pharmacy, “Victor Babeș” University of Medicine and Pharmacy, Eftimie Murgu Sq. no. 2, 300041 Timișoara, Romania; 7 Department VIII—Neuroscience, Discipline of Medical Deontology. Bioethics, Faculty of Medicine, “Victor Babes” University of Medicine and Pharmacy, Eftimie Murgu Sq. no. 2, 300041 Timisoara, Romania; ramona.parvanescu@yahoo.com; 8 Department of Analytical Chemistry, Faculty of Pharmacy, “Victor Babeș” University of Medicine and Pharmacy, Eftimie Murgu Sq. no. 2, 300041 Timișoara, Romania; fborcan@umft.ro; 9 Department of Plastic and Reconstructive Surgery, Faculty of Medicine, “Victor Babeș” University of Medicine and Pharmacy, Eftimie Murgu Sq. no. 2, 300041 Timișoara, Romania; zcrainiceanu@gmail.com

**Keywords:** *Melissa officinalis* aqueous extract, DPPH, FTIR, LC-MS, cell viability, SKH-1 hair-less mice, skin parameters, angiogenesis, CAM assay

## Abstract

Melissa officinalis (MO) is a medicinal plant well-known for its multiple pharmacological effects, including anti-inflammatory, anticancer and beneficial effects on skin recovery. In this context, the present study was aimed to investigate the in vitro and in vivo safety profile of an MO aqueous extract by assessing cell viability on normal (HaCaT—human keratinocytes) and tumor (A375—human melanoma) cells and its impact on physiological skin parameters by a non-invasive method. In addition, the antioxidant activity and the antiangiogenic potential of the extract were verified. A selective cytotoxic effect was noted in A375 cells, while no toxicity was noticed in healthy cells. The MO aqueous extract safety profile after topical application was investigated on SKH-1 mice, and an enhanced skin hydration and decreased erythema and transepidermal water loss levels were observed. The in ovo CAM assay, performed to investigate the potential modulating effect on the angiogenesis process and the blood vessels impact, indicated that at concentrations of 100 and 500 µg/mL, MO aqueous extract induced a reduction of thin capillaries. No signs of vascular toxicity were recorded at concentrations as high as 1000 μg/mL. The aqueous extract of MO leaves can be considered a promising candidate for skin disorders with impaired physiological skin parameters.

## 1. Introduction

The medicinal aromatic plant *Melissa officinalis* L., known as lemon balm, common balm or sweet balm, has a long tradition in different regions around the world in various ethno-medical treatments as a sedative, for memory improvement, as an antiviral, antispasmodic and antibacterial substance and as a carminative [1,2,3,4,5,6,7].

*Melissa officinalis* L. is an aromatic and perennial herb, with honey-producing capacity, which belongs to the *Lamiaceae* family [8]. People frequently consume *Melissa officinalis* herbal tea to ensure a good digestion, to reduce gastrointestinal disorders, to avoid sleep disturbance and for its antispasmodic properties [9,10]. Furthermore, many research studies have shown that MO extract possesses promising antitumor potential in various human cancer cell lines [11,12,13,14,15,16]. Cancer, a highly aggressive and heterogenic disease, is considered one of the causes for the elevated mortality rates worldwide [17]. Although notable advances were made in the therapeutic management of cancer, new efficient treatments with minimal side effects are highly demanded. Natural products are the source of many Food and Drug Administration (FDA)-approved anticancer drugs, and they still represent a major alternative for research [18,19]. Cutaneous melanoma, one of the most common cancers among young adults, especially in young women, has an incidence rate steadily increasing globally [20,21]. Due to the high resistance developed by melanoma cells following the administration of cytotoxic agents used as treatment for metastatic melanoma [22], finding novel anticancer compounds that overcome these liabilities has become an urgent need. An intact skin barrier ensures a strong defense against different environmental, chemical, mechanical and microbial factors, whereas a dysfunctional one might be the starting point for various skin disorders [23]. To that end, the products for skin integrity maintenance that also exhibit potential antitumor activity might be considered promising alternatives for the therapy of skin pathologies. Thus, plant extracts rich in bioactive compounds are highly investigated in order to find alternatives to conventional chemical treatments. Different species of *Lamiaceae* family were assessed in this regard, due to their confirmed phytotherapeutic potential [24]. 

The complex chemical composition plant extracts, including: hydroxycinnamic acids (rosmarinic, *p*-coumaric, caffeic, chlorogenic and ferulic acids), triterpenes (ursolic and oleanolic acids), essential oils rich in terpenoids (citral, citronellal, geraniol, nerol, linalool, farnesyl acetate, humulene, β-caryophyllene and eremophilene), tannins and flavonoids (glycosides of luteolin, quercetin, apigenin and kaempferol) represented the main reason for the use of different types of extracts or pure volatile oils for the prevention and/or treatment of an increased number of illnesses [25,26,27,28,29,30,31]. The MO activity is related to the high concentration of phytocompounds that exert protective properties against oxidative stress, a key player in the aging process and the onset of degenerative diseases [32]. The complex chemical composition of MO was associated with beneficial effects in several skin disorders [33]. Ramanauskienė and co-workers have designed a pharmaceutical semisolid formulation with *Melissa officinalis* extract to investigate the protective effect of one of the main active phytocompounds (rosmarinic acid) on skin cells both in normal conditions and under oxidative stress. The results obtained showed that rosmarinic acid reduced the amount of intracellular ROS (reactive oxygen species) in a concentration-dependent manner in human keratinocytes and enhanced cell viability under oxidative stress conditions [34]. More than a decade ago, MO has been used as a folk remedy to treat eczema, insect bites, wounds and even for treating the pruritic skin disorder, using an MO oil-based formulation [35]. Zhou and co-workers were the first group of researchers who demonstrated the immunoregulatory potential of MO oil in alleviating atopic dermatitis via interfering with the Th2 / Th1 cell activation. The MO oil efficiency on atopic dermatitis-like immune alteration and skin lesions was evaluated using BALB/c mice. Following the study, the researchers could not specify which of the MO oil chemical compounds contributed to the alleviation of atopic dermatitis-like immunologic and skin alterations in mice [36]. In a study conducted by Ippoushi and co-workers [37], the methanolic extract of MO leaves showed the strongest in vitro inhibitory effect on hyaluronidase, which is known to be related to inflammation and involved in tumor cells migration. 

The present investigation was undertaken to evaluate the efficacy and safety use of an aqueous extract of MO leaves and the possible alterations or improvements on female SKH-1 hairless mice skin. In order to complete these results, the study focused on the evaluation of an aqueous extract of MO leaves as a viability modulator in A375 human melanoma tumor cell lines and in a normal cell line, HaCaT, immortalized human keratinocytes. The physico-chemical profile of the aqueous MO leaves extract was also established. In addition, the antioxidant activity of the aqueous extract of MO leaves was assessed next to its antiangiogenic potential in ovo, a valuable approach to control tumor growth and invasiveness in cancer.

## 2. Results

### 2.1. Antioxidant Activity Assessment

**Figure 1:**
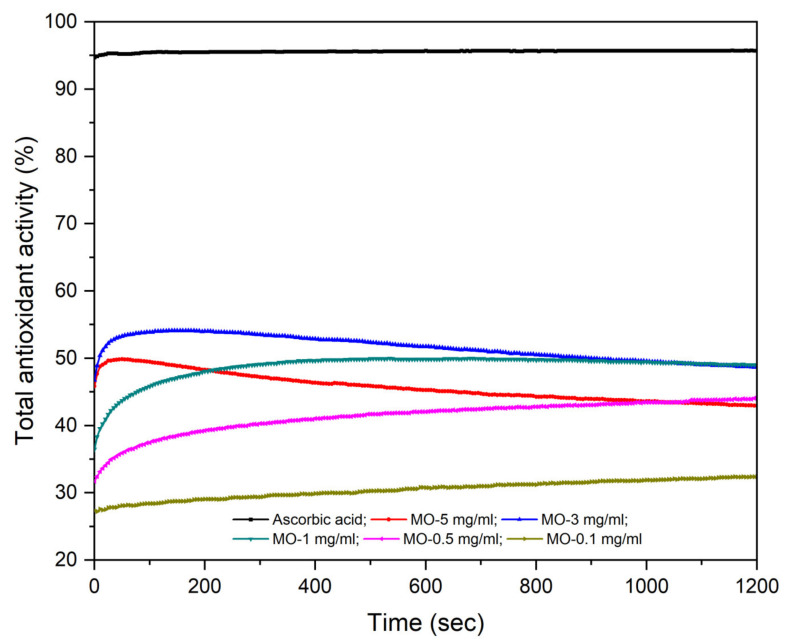
Time-dependent antioxidant activity of the aqueous extract of MO leaves.

The antioxidant activity (AOA) of ascorbic acid (employed as standard) compared to an aqueous extract of MO leaves in different concentrations (MO 5 mg/mL; 3 mg/mL; 1 mg/mL; 0.5 mg/mL and 0.1 mg/mL) are shown in Figure 1. The samples were analyzed in a time-dependent manner, in a continuous mode, for 20 min, in order to establish the time and the reaction speed for DPPH radical consumption by the antioxidants present in the aqueous extract of MO leaves. All five aqueous extracts of MO leaves showed an antioxidant activity above 30%.

At the end of the reaction, the highest concentration of aqueous extracts of MO leaves, 5 mg/mL, exhibited the highest AOA (49.11%) compared with the other concentrations tested, while the lowest values were recorded for the aqueous extract of MO leaves at 0.1 mg/mL (32.26%). In all tested samples, the compounds with antioxidant properties reacted with the DPPH radical throughout the recording time of the analysis, and it can be said that at the end of reaction, an equilibrium was reached only for the aqueous extracts of MO leaves at 5 mg/mL, 3 mg/mL and 0.5 mg/mL. In the case of the 1 mg/mL and 0.1 mg/mL aqueous extracts of MO leaves, the antioxidants contained in the samples react with the DPPH radical throughout the recording time of the analysis; however, at the end of the reaction, equilibrium was not reached. In this case, a longer analysis time would have been necessary. Figure 1 shows that 3 mg/mL and 0.5 mg/mL aqueous extracts of MO leaves had an identical kinetics reaction, indicating that both extracts reacted with the DPPH radical in the first 100 s; afterward, an increase was recorded in the absorbance of the two samples, more visible for the aqueous extract of MO leaves at 0.5 mg/mL. This can be explained by the fact that the solid MO leaves powder from the two samples may not have been completely dispersed in distilled water. In the case of the other tested concentrations, a decrease was recorded in the absorbance values throughout the analysis, which led to an increase in antioxidant activity. According to these results, the antioxidant activity of all tested samples was concentration-dependent, in compliance with the inhibition percentage (% inhibition) of ascorbic acid and of the tested concentrations of aqueous extracts of MO leaves. The EC_50_ values for ascorbic acid (EC_50_ = 4.07 × 10^−4^ ± 3.33 × 10^−4^ mg/mL) and for aqueous extract of MO leaves (EC_50_ = 4.3113 ± 1.30671 mg/mL) were also calculated, which indicate the effective concentration necessary to obtain 50% of DPPH degradation.

### 2.2. FTIR Spectra

Using a Fourier-transformed infrared (FT-IR) analysis, the identification of functional groups (Table 1) resulted from the aqueous extract of dried MO leaves. The analysis is based on the presence of peaks at different wavenumbers, in the region of IR radiation. 

The FT-IR analysis recorded strong absorption bands at 3431, 2974, 1636, 1400 and 1076 cm^−1^. The first important band is located at 3431 cm^−1^, a strong, wide and well-defined band. This band can be attributed to the O-H stretching vibration (hydroxyl groups (H-bonded)) present in water or in flavones, contained in the aqueous extract of dried MO leaves. The band located at 2974 cm^−1^ can be assigned to the O-H stretching in acid functional groups. The strong band at 1636 cm^−1^ indicates that the C=C group is present in the alkenes, but it could also be assigned to the C=O stretching vibration of carbonyl functional groups or to amide functional groups. At 1400 cm^−1^ is the absorption band corresponding to –C-H bending functional group in alkane. The presence of a band between 1370 and 1250 cm^−1^ suggests the presence of the C-O stretching vibration in lactone or in flavones. At 1076 cm^−1^, the well-defined band could be attributed to the C-O vibration in ester functional groups and to the C-O stretching in secondary cyclic alcohols. The region between 650 and 910 cm^−1^ is specific for the out-of-plane bending vibration of ring =C-H bonds present in the aromatic compounds, which in this case could be specific for aromatic bicyclic monoterpenes.

### 2.3. LC-MS Assessment of the Aqueous Extract of Melissa officinalis Leaves 

The aqueous extract of MO leaves was subjected to LC-MS analysis and screened for 18 polyphenols, which enabled the identification/quantification, consistent with their Rt and *m*/*z* values, of 7 polyphenolic phytocompounds: gentisic acid, *p*-coumaric acid, apigenin, chlorogenic acid, caffeic acid, rutin and ferulic acid expressed in ng/mg d.w. (Table 2). The elution of all components was achieved in about 40 min. Obtained results indicated that apigenin was the most abundant polyphenolic quantified compound (Table 2). Gentisic acid and *p*-coumaric acid were also identified, but they exhibited low concentrations, which fell below the limit of quantification.

### 2.4. MO Aqueous Extract Effect on Cells’ Viability Is Cell-Type Dependent

**Figure 2:**
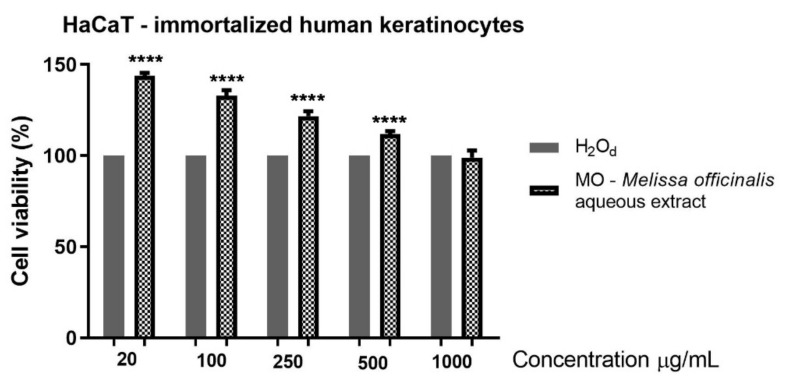
Cell viability assessment of an aqueous extract of MO leaves (20, 100, 250, 500 and 1000 μg/mL) in HaCaT cells at 24 h post-stimulation by means of Alamar blue assay. The results are expressed as a cell viability percentage (%) normalized to cells stimulated with distilled water. The data represent the mean values ± SD of three independent experiments. A one-way ANOVA analysis was applied to determine the statistical differences compared with distilled water-treated cells followed by Tukey’s post-test (**** *p* < 0.0001).

The effect of an aqueous extract of MO leaves was evaluated by means of Alamar Blue technique on a non-tumor, HaCaT, and on a tumor, A375 human melanoma cell lines. Five concentrations were tested: 20, 100, 250, 500 and 1000 μg/mL. The results obtained on HaCaT cells after 24 h stimulation indicate that an aqueous extract of MO leaves led to a significant increase in cell proliferation, and even at the highest dose tested (1000 µg/mL), no toxicity was observed (Figure 2).

**Figure 3:**
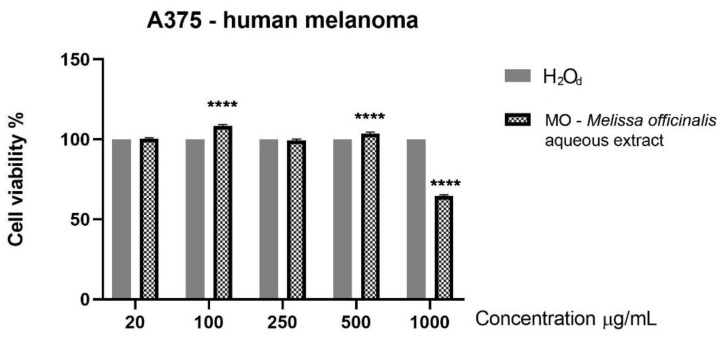
Cell viability assessment of aqueous extracts of MO leaves (20, 100, 250, 500 and 1000 μg/mL) in A375 human melanoma cells at 24 h post stimulation by means of Alamar blue assay. The results are expressed as cell viability percentage (%) normalized to cells stimulated with distilled water. The data represent the mean value ± SD obtained from three independent experiments. One-way ANOVA analysis was applied to determine the statistical differences compared with distilled water-treated cells followed by Tukey’s post-test (**** *p* < 0.0001).

The response of A375 human melanoma cells to an aqueous extract of MO leaves after 24 h of stimulation consisted of a decrease of tumor cells’ viability (approximately 64% vs. control) that was observed only at the highest tested concentration, 1000 μg/mL, while the other tested concentrations did not reduce the cells’ viability (Figure 3). Based on the data obtained, it can be stated that an aqueous extract of MO leaves exerted a cytotoxic effect at the highest dose used in A375 cells and proved to be safe when tested on the healthy cells, i.e., HaCaT keratinocytes.

### 2.5. Tumor Cells Morphology 

A375 melanoma cells morphology was evaluated at 24 h post stimulation with aqueous extracts of MO leaves extracts (20 µg/mL–1000 µg/mL). The images indicate that at low concentrations, the confluence was not affected being similar to the control group, but at the highest dose tested (1000 µg/mL), treatment with the aqueous extract of MO leaves induced significant morphological changes. Some of the tumor cells became round and started to detach. The cells stimulated with the solvent (distilled water) were adherent to the culture plate and did not display changes in their morphological aspect. These results are in accordance with the data obtained in the cell viability assay.

### 2.6. Chorioallantoic Membrane Assay

**Figure 4:**
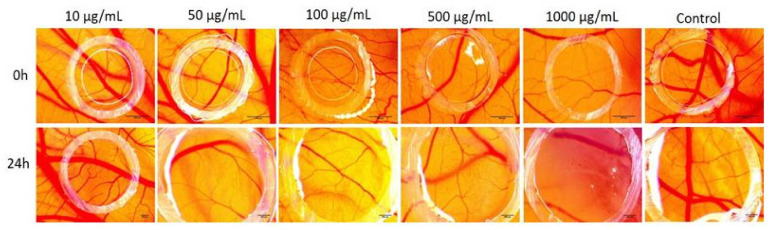
Stereomicroscopic images regarding the effects of the aqueous extracts of MO leaves on the chorioallantoic membrane assay. Evaluation was assessed before application (0 h) and at 24 h after the application of samples (five concentrations of MO aqueous extracts: 10, 50, 100, 500 and 1000 µg/mL) and control (distilled water).

The aqueous extract of MO leaves was tested on the chorioallantoic membrane in order to evaluate the potential implications in the process of angiogenesis (Figure 4). The application of the samples started on the 7^th^ embryonic day of development, considering that between 7–11 EDD is the period when the endothelial cells are highly mitotic, and testing in this interval is indicative for a potential effect on the inhibition of the angiogenesis process. Multiple concentrations were evaluated and a dose-related effect on the inhibition of the blood vessel was observed, compared to solvent control, which did not influence normal angiogenesis.

After 24 h of contact with the samples, inside the application ring, up to a concentration of 50 µg/mL, there were no modifications regarding the normal angiogenic process. The higher concentrations showed an increasing effect on the formation of new capillaries, *Melissa officinalis* extracts in concentration of 100 and 500 µg/mL, leading to a lower number of thin capillaries, lacking numerous interconnecting points. The highest concentration 1000 µg/mL induced the most visible reduction of blood vessels inside the application ring. No signs of toxicity upon the blood vessel functionality were noted during the experimental process.

### 2.7. Skin Biophysical Parameters Assessment

**Figure 5:**
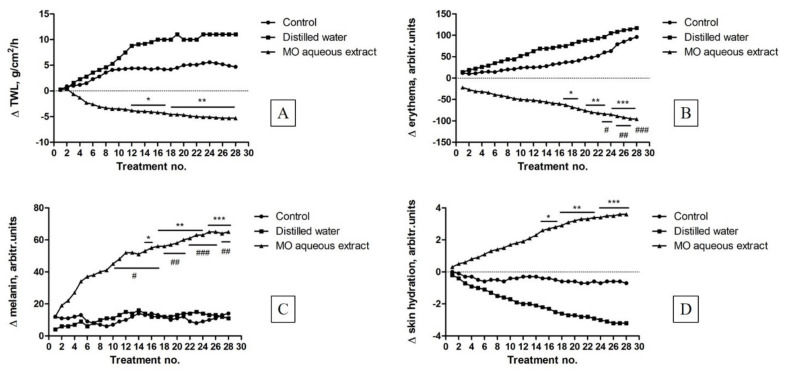
Comparative evolution of skin biophysical parameters. (**A**) Transepidermal water loss (for MO aqueous extract vs. distilled water * *p* < 0.05; ** *p* < 0.001). The statistical differences were determined using a two-way ANOVA analysis followed by a Bonferroni post-test; (**B**) Erythema (for MO aqueous extract vs. distilled water * *p* < 0.05; ** *p* < 0.001; *** *p* < 0.001; for MO aqueous extract vs. Control # *p* < 0.05; ## *p* < 0.001; ### *p* < 0.001). The statistical differences were determined using a two-way ANOVA analysis followed by a Bonferroni post-test; (**C**) Melanin (for MO aqueous extract vs. distilled water * *p* < 0.05; ** *p* < 0.001; *** *p* < 0.001; for MO aqueous extract vs. Control # *p* < 0.05; ## *p* < 0.001; ### *p* < 0.001). The statistical differences were determined using a two-way ANOVA analysis followed by a Bonferroni post-test; (**D**) Skin hydration (for MO aqueous extract vs. distilled water * *p* < 0.05; ** *p* < 0.001; *** *p* < 0.001). The statistical differences were determined using a two-way ANOVA analysis followed by a Bonferroni post-test.

In order to evaluate the safety profile of the aqueous extract of MO leaves as treatment for different skin homeostatic parameters changes, an in vivo experiment was performed using female SKH-1 hairless mice skin as a common model for this aim. Our data indicated that an aqueous extract of MO leaves at a dose of 5 mg/mL elicited significant changes in the main murine skin parameters, reported as positive aspects: an increase of the transepidermal water loss was found in the case of the mice used as control and those treated with the solvent (distilled water) (Figure 5A), while decreased values of this parameter were recorded in the group of mice treated with an aqueous extract of MO leaves.

Figure 5B presents the main skin parameter, which can be used to predict the safety of the aqueous extract based on MO leaves: the erythema level. As described for the previous parameter, relevant differences between the group treated with an aqueous extract of MO leaves and the other groups were also observed in terms of erythema. The erythema index presented a noticeable decrease due to the active ingredients contained in the aqueous extract based on MO leaves that seem to have a beneficial potential to heal skin injuries.

Melanin is a natural pigment that is involved in the skin and hair coloring mechanisms; its evolution in every mice group is depicted in Figure 5C. A significant difference between the mice treated with an aqueous extract of MO leaves and the other mice was observed. In the case of SKH-1 mouse, this parameter is correlated with skin erythema.

An evolution of skin hydration following application of the aqueous extract of MO leaves was also verified in mice (Figure 5D). The increased values of mice skin hydration recorded after each treatment applied represent a confirmation of the beneficial potential of MO aqueous extract obtained from leaves, as skin moisture is a mark of healthy skin.

## 3. Discussion

The present study was conducted to evaluate the phytochemical and biological profile of an aqueous extract of MO leaves by assessing the antioxidant activity correlated with other relevant bioactivities: the in vitro cytotoxicity, the impact on the angiogenesis process in ovo and on physiological skin parameters in vivo. There are some important factors which determine the antioxidant activity of plants, for example the type and the part of the plant (leaves, stems, flower or seeds), the habitat and the location, the climatic conditions or soil characteristics. Other important factors refer to the solvent used in the extraction process (water, alcohol, organic mixtures, etc.), the extraction method and the extraction time [38]. Moreover, the harvesting period of plant material could change the extract’s quality. Regarding the solvent used for extraction, water is also used as an extracting agent due to the simplicity of the experimental protocol and its unique properties as a solvent, which can be changed by temperature and pH.

The antioxidant activity of the aqueous extract of MO leaves was monitored using the DPPH radical inhibition method. It was shown that all tested concentrations possessed antioxidant activity in a concentration-dependent manner. According to previous studies, a low polar extraction media of lemon balm leaves confers good antioxidant and antitumoral activities [39,40,41]. In this study, we demonstrated that even a strong polar extract of MO leaves exhibited good antioxidant properties. Kwon et al. [42] demonstrated that the aqueous extract of MO exhibited better antioxidant properties when compared with the ethanolic one. The authors achieved a DPPH radical inhibition activity of 67.6% for the aqueous MO extract, even if the total phenolic content was lower as compared to the ethanolic extract investigated. Our data are comparable to those obtained by Kwon and co-workers (49.11% vs. 67.6%) with minor differences that might result from the extract preparation protocol. In the present study, the ratio between the MO dried powder and distilled water was 1 g:100 mL, whereas in the study conducted by Kwon and co-workers, the ratio was 5 g:100 mL.

In the present study, the antioxidant activity was also expressed as EC_50_ value, yielding an EC_50_ = 4.3113 mg/mL for the aqueous extract based on MO leaves. Our data are comparable to the data present in the literature, showing a slightly higher antioxidant potential. For instance, Kamdem and co-workers showed that *Melissa officinalis* ethanolic extract has the ability to scavenge the DPPH radical directly proportional to the concentration leading to an IC_50_ of 48.76 ± 1.94 μg/mL [43]. In the present study, the aqueous extract of MO leaves exhibited a higher value of AOA as compared to the AOA of MO leaves ethanolic extracts obtained by other researchers. Mohamadi et al. [44] showed that the antioxidant properties of a methanolic extract from *Melissa officinalis* on the stability of soybean oil indicated a value of 0.043 mg/mL necessary to degrade 50% of DPPH radical. Pereira and co-workers studied the antioxidant potential of aqueous, ethanolic and methanolic extracts obtained from aerial parts of sweet balm. The study concluded that all type of extracts present important radical scavenging potential [45]. The main remark is that the antioxidant potential of different plant extracts makes them proper and safe candidates as anticancer agents, since they have the ability to scavenge free radicals, and consequently, protect cells from their hazardous effects [46].

The results of the present study provided by the FT-IR spectroscopy analysis were interpreted in accordance with the Characteristic IR Absorption Frequencies of Organic Functional Groups (Characteristic of IR Absorption Frequencies of Organic Functional Groups). The results regarding the functional groups from aqueous extract of the dried MO leaves are in agreement with data from the literature [47,48,49,50,51,52,53,54]. The FT-IR analysis performed in the present study demonstrated that all the bands were assigned to a vibrational mode and the active compounds identified were assigned to a specific wavenumber value (Table 1). The most important band, located at 3431 cm^−1^, assigned to the O-H stretching vibration (hydroxyl groups (H-bonded)), could be attributed either to water trace from the aqueous extract of MO leaves or to flavones such as apigenin or rutin. The O-H stretching assigned to acid functional groups recorded at 2974 cm^−1^ could be attributed to gentisic acid, caffeic acid or ferulic acid. The C=C and C=O groups recorded at 1636 cm^−1^ could be attributed to hydroxycinnamic acids (*p*-coumaric acid), chlorogenic acid, ferulic acid or rutin. The -C-H group recorded at 1269 cm^−1^ and the C-O group recorded at 1076 cm^−1^ could be attributed to flavones like apigenin, rutin or even to chlorogenic acid, since it is the ester of caffeic acid, due to the fact that flavonoids/flavones contain ester-like functional groups in their structure.

Due to the fact that globally, there is a growing need to know precisely the identity and the content of each compound in the plant material, an LC-MS analysis of polyphenols was performed. Phenols are highly effective antioxidants contained in plants represented by a great variety of biological active compounds. In accordance with the FT-IR analysis, the biological active compounds contained in an aqueous extract of MO leaves were: flavonoids (apigenin, rutin), hydroxycinnamic acids (*p*-coumaric acid, caffeic acid, chlorogenic acid and ferulic acid) and dihydroxybenzoic acids (gentisic acid) (Table 2). Gentisic acid and *p*-coumaric acid were below the limit of quantification. Various research studies and herbal drug quality monographs report the identification of several polyphenols from aqueous extract of MO, including rosmarinic acid [55,56,57,58]. The absence of rosmarinic acid in the present study may be due to a variety of reasons ranging from climate and geography to differences regarding the extraction strategies and solvents. Rutin, the second major identified compounds in this study, has already been reported to be present in the aqueous extract of MO, but in a lower amount [59]. Regarding the presence of chlorogenic, caffeic and ferulic acid in aqueous extract of MO leaves, other researchers have found larger quantities than those reported in this study [58,59]. A study conducted by Lin et al. [60] reported that the ethanolic extract of freeze-dried MO leaves had a higher phenolic acids content than the ethanolic extract of hot air-dried MO leaves. These authors also found that gentisic acid and apigenin were not detected and that *p*-coumaric acid content was detected only as 0.32 mg/g of dried extract. Instead, in the present study, apigenin content was 38.72 ng/mg d.w. in the aqueous MO leaves extract.

Another analysis that was conducted in the present study consists of the cell viability assessment of the aqueous extract of MO leaves in healthy HaCaT keratinocytes and in A375 human melanoma cells. The data obtained indicated that the aqueous extract elicited a significant increase in keratinocytes proliferation. At the highest dose tested (1000 µg/mL), HaCaT cells viability was not affected by the MO extract treatment. In a previous study, Moacă et al. [14] showed that an ethanolic extract of *Melissa officinalis* leaves reduced HaCaT cells’ viability at 24 h post-stimulation at the highest concentrations tested, 500 and 1000 µg/mL. Based on the results obtained, it can be stated that the aqueous extract of MO leaves is less cytotoxic than the ethanolic one on the human keratinocytes and could be considered a good candidate for in vivo evaluation. Regarding the effect on the melanoma cells, the aqueous extract of MO leaves exerted a significant reduction of cells’ viability percentage only at the highest tested dose. At low concentrations, at 100 and 500 µg/mL, the aqueous extracts of MO leaves increase A375 tumor cells viability. This effect can be due to a possible hormetic effect that produced a stimulation at low doses and an inhibition at high doses. Numerous research papers have documented a hormetic response in various fields, including carcinogenesis and toxicology [61]. Other types of extracts (methanolic, ethanolic) proved to possess higher cytotoxic effects on various tumor cell lines [14,62,63], but also affected the healthy cell lines. This is the case of an MO ethanolic extract tested on MDA-MB-231 breast adenocarcinoma cells for 24 h in the same concentrations as the ones chosen in this study [14]. The ethanolic extract induced a dose-dependent cytotoxic effect in breast cancer cells, but a cytotoxic effect was also visible in the case of healthy HaCaT keratinocytes. Based on our cell viability results, it could be stated that the aqueous extract of MO leaves exerts a cell type-dependent cytotoxicity oriented towards A375 human melanoma cells, whereas HaCaT cells viability was not affected.

Lemon balm aqueous extracts were reported to elicit antitumor effect in various cancer cell lines, namely breast adenocarcinoma [12], colon cancer [64], prostate cancer [65] and others. The existent scientific data do not offer sufficient information regarding the effect of aqueous lemon balm extracts on melanoma.

In order to assess the potential antiangiogenic effect of the aqueous extract of MO leaves, we used the chick embryo chorioallantoic membrane (CAM) assay, an in vivo method with several advantages, such as as low cost, time, simplicity and reproducibility [66]. The CAM assay is a promising alternative method, designed both to reduce the number of animals used as well as to obtain preliminary data for in vivo animal models. This method is an internationally validated method proposed by the Interagency Coordinating Committee on the Validation of Alternative Methods [67]. The results regarding the antiangiogenic potential showed a dose-related effect on the inhibition of the blood vessel formation. At 50 μg/mL, no modification was observed regarding the angiogenic process, even after one day of contact. By increasing the concentration of the aqueous extract (1000 μg/mL), a visible reduction of blood vessels inside the application ring was observed. No signs of toxicity were signaled on the CAM highly vascularized mucosal tissues, confirming the in vitro results on normal HaCaT cells at the highest tested concentration (1000 μg/mL).

*Melissa officinalis* extracts were previously reported for a potential antiangiogenic effect. Studies were conducted to explore the relation between angiogenesis process and obesity. In vitro assays on HUVEC cells and in vivo experiments on mice confirmed a reduction of angiogenesis and blood vessel density both in aqueous and ethanolic extracts from lemon balm leaves, thus being responsible for the modulation of adipogenesis and showing no signs of cytotoxicity [68,69]. Our results indicate that higher concentrations of the aqueous extract from MO leaves reduce the high angiogenic process of the chorioallantoic membrane, therefore representing interesting candidates for the limitation of tumor growth and invasion.

To complete the safety profile of an aqueous extract of MO leaves, its impact on different physiological skin parameters was also assessed by non-invasive methods using SKH-1 hairless mice.

The non-invasive procedures revealed small changes of the measured parameters in the case of control group, but these changes are an important clue to establish the sensitivity of mice skin. Mouse skin presents several differences in thickness and in the number of cells but not the layers of cells in dermis and epidermis as compared to human skin [70]. Still, mouse skin is considered a relevant model for the evaluation of compounds that act on skin by the means of non-invasive measurements [71]. The in vivo results obtained in this study indicate the benefits of aqueous MO leaves extract at skin level characterized by reduced values of erythema and enhanced values of skin moisture (both parameters are significantly modified compared to the other groups). The anti-inflammatory activity of lemon balm was also mentioned in the literature [34]. Ramanauskiene and co-workers designed gels with lemon balm extract and investigated the effect of rosmarinic acid on skin cells in normal conditions and under oxidative stress. The authors demonstrated in HaCaT cells that in oxidative stress conditions, rosmarinic acid released from gels with lemon balm extract, reduced intracellular ROS amounts, increased cell viability and protected cells from the damage caused by H_2_O_2_ [34]. The function of these antioxidants (ROS involvement) and how they improved skin quality was also the main observation in the present work.

It is worth to mention the slight increase of the melanin level observed to the group of mice treated with the aqueous extract based on MO leaves. These data are correlated to erythema values. The main pigments that influenced these parameters are hemoglobin, bilirubin and mostly melanin. Erythema is associated with some skin pathological aspects, including skin injury, inflammation or in some cases infection [71]. The aqueous extract protects and hydrates the mice skin. Similar results have been obtained by a Japanese research group, who have studied the anti-inflammatory effects of a lemon balm oil on BALB/c mice with induced atopic dermatitis on the dorsal skin [36]. Atopic dermatitis is also correlated with skin thickness and an increase of TEWL values. The present work presented an important elevation of skin hydration and an improvement in the skin integrity barrier following extract application.

Elevated TEWL values indicated skin abnormalities. Skin hydration indicated epidermal function. A high content and non-high variable data represent proper epidermal function. The hydration level is generally an indicator of proper epidermal function. It can be determined non-invasively by using a Corneometer. Hydrolipid film is an indicator for healthy skin [71].

## 4. Experimental Section

### 4.1. Materials

#### 4.1.1. Chemical/Reagents

Chemical compounds were purchased from the following sources: ethanol 96% (*v/v*) and distilled water from Chemical Company SA (Iasi, Romania); 2,2-diphenyl-1-picrylhydrazyl (DPPH) (Batch No: # STBF5255V) from Sigma–Aldrich (Steinheim, Germany) and the ascorbic acid from Lach-Ner Company (Prague, Czech Republic). In order to perform LC-MS experiments, methanol (99.9% purity) and acetic acid (99.9% purity) were purchased from Merck (Darmstadt, Germany). Standard poly-phenolic compounds, rosmarinic acid, caftaric acid, gentisic acid, chlorogenic acid, caffeic acid, *p*-coumaric acid, ferulic acid and sinapic acid, hyperoside, isoquer-citrin, rutin, myricetin, fisetin, quercitrin, quercetol, luteolin, kaempferol and apig-enin, were purchased from Sigma–Aldrich (Steinheim, Germany). Ultrapure deionized water was provided by the MiliQ system, Milli-Q^®^ Integral Water Purification System (Merckmilipore, Darmstadt, Germany).

#### 4.1.2. Cell Lines and Culture Media

The tumorigenic cell line—A375 skin achromic human melanoma cells—was obtained from American Type Culture Collection (ATCC^®^, cat no. CRL-1619™) (LGC Standards GmbH, Wesel, Germany) and the normal cell line, immortalized human keratinocytes—HaCaT—was received as a gift from the University of Debrecen. The culture media and reagents specific for cell culture were: Dulbecco’s modified Eagle Medium (DMEM), high glucose, trypsin/EDTA solution, phosphate saline buffer (PBS), penicillin/streptomycin solution, fetal bovine serum (FBS), Trypan blue and Alamar blue solutions, supplied by Sigma-Aldrich (Munich, Germany) and Thermo Fisher Scientific (Inc., Waltham, MA, USA).

The cells were cultured in DMEM supplemented with 10% fetal calf serum (FCS). To avoid culture contamination, an antibiotic mixture (penicillin and streptomycin 100 U/mL) was also added. During the experiment, the cells were maintained in the incubator in a humified atmosphere at 37 °C and 5% CO_2_.

#### 4.1.3. Plant Material

*Melissa officinalis* leaves were collected from the Botanical garden of “Victor Babes,” University of Medicine and Pharmacy Timisoara (Romania, 45°47′58″N latitude 21°17′38″E longitude). The material used in the study was identified and certified by Prof. Dr. Diana S. Antal, from the Pharmaceutical Botany Department, Faculty of Pharmacy, “Victor Babes” University of Medicine and Pharmacy Timisoara and received the reference number: 125/20016. A specimen sample was kept at the herbarium of the mentioned department, in a temperature-controlled place (22–25 °C and 30–40% relative humidity). The harvested leaves were air dried at room temperature (25 °C) and stored in proper conditions (in dark flasks at laboratory temperature and normal atmosphere).

### 4.2. Animals

In order to evaluate the effect of an aqueous extract of MO leaves on skin, an in vivo experiment was performed using nine female SKH-1 hairless mice, 11–12 weeks old (weight = 20 ± 2 g), purchased from Charles River Laboratory (Budapest, Hungary). The mice were kept in specific cages under the following controlled conditions: temperature 24 ± 1 °C, humidity above 55% and a 12-h light/dark cycle (7:00 a.m. to 7:00 p.m.), in accordance with the Guide for the Care and Use of Laboratory Animals (1996, published by National Academy Press). Food was available ad libitum and water was provided and could be freely accessed, as recommended by the European Directive 2010/63/EU and the national law 43/2014. There was a one-week adaptation period, after which the mice were divided into three groups (three mice/group): group 1: control group (no intervention), group 2: mice treated with the solvent (distilled water) and group 3: mice treated with the aqueous extract of MO leaves. The in vivo experiment was performed according to the following protocol: the aqueous extract of MO leaves (5 mg/mL) was topically applied (40 μL) on their dorsal skin every two days. The same procedure was applied to the group treated with the solvent, distilled water (40 µL). The experiment has been approved by the Bioethical Committee of “Victor Babes,” University of Medicine and Pharmacy Timisoara (protocol code no. 2/10.02.2020), and respected the international regulations.

### 4.3. Methods

#### 4.3.1. Preparation of an Aqueous Extract of MO Leaves 

**Figure 6:**
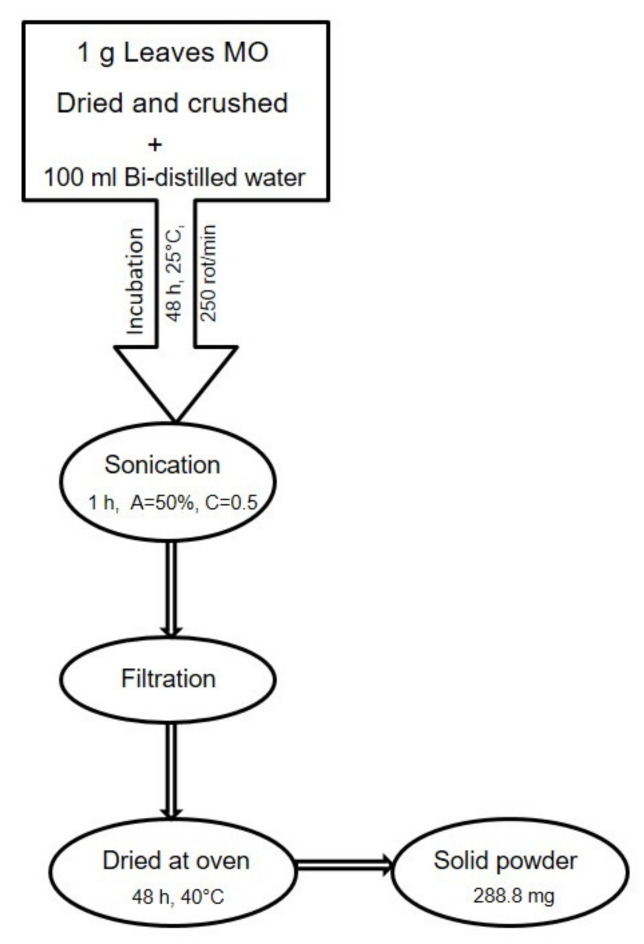
Schematic protocol of the method used to obtain the aqueous extract of dried *Melissa officinalis* leaves.

The aqueous extract of *Melissa officinalis* leaves was obtained according to the Skooti et al. protocol, slightly modified [54]. Briefly, 1 g of dried and crushed lemon balm leaves was mixed with 100 mL distilled water, and the mixture was kept in an incubator with an orbital stirrer (ES20/60 Biosan, Riga, Latvia) for 48 h at 25 °C and 250 rot/min. After the incubation time, the mixture was sonicated for 1 h at 50% amplitude (750 W, Q Sonica Sonicators, Newtown, CT, USA). The sonication step was followed by filtration of the aqueous extract, using Whatman membrane filters nylon 0.45 μm, 30 mm (Sigma–Aldrich; Merck KGaA, Darmstadt, Germany) and dried at 40 °C in an oven (POL-EKO Aparatura, Wodzisław Śląski, Poland) until a solid powder was obtained. The concentrations tested, consisting of the aqueous extract of MO leaves, were obtained by dissolving the solid extract powder in distilled water. The schematic protocol is depicted in Figure 6.

#### 4.3.2. Physicochemical Characterization of an Aqueous Extract of MO Leaves 

##### Antioxidant Activity Assay

The antioxidant activity (AOA) assay of aqueous extracts of MO leaves was determined by the DPPH (2,2-diphenyl-1-picrylhydrazyl) technique, which was developed and detailed in a previous study conducted by our group [14]. An ethanolic solution of 1 mM of DPPH was prepared and kept in a dark place, at 4 °C, until use. The absorbances values of the reaction mixtures were measured at a wavelength of 517 nm using an UviLine 9400 Spectrophotometer from SI Analytics (Mainz, Germany), until an equilibrium was obtained. The analysis was carried out in triplicate and the results were compared with the results obtained for ascorbic acid in ethanol solution (0.4 mg/mL), used as etalon.

The inhibition percent (%AOA) was calculated with the following formula:

(1) $$\%AOA=\left[\frac{A_{control}-A_{extract}}{A_{control}}\right]\times 100$$

where AOA: antioxidant activity of the analyzed aqueous extracts of MO leaves (%); A_control_: the absorbance of the mixture, without extract (distilled water and DPPH radical of 1 mM); A_extract_: the absorbance of the mixture, with extract (aqueous extract of MO leaves + DPPH radical of 1 mM);

The antioxidant activity of the aqueous extracts of MO leaves was expressed as EC_50_ value, which is calculated by non-linear regression, using Origin 2020b software. EC_50_ represents the concentration of the antioxidant compounds contained in the extract, required to degrade the DPPH radical by 50%.

##### FTIR investigation of MO solid powder leaves extract

In order to identify the functional groups of the main active compounds which are present in solid powder of the leaf extract, the Fourier-Transformed Infrared Spectroscopy was employed. The infrared spectrum was recorded in the range 400–4000 cm^−1^, with a resolution of 4 cm^−1^, with a Shimadzu Prestige-21 spectrometer (Duisburg, Germany). The KBr pellet method was applied. Depending on the appearance of a peak at a certain wavenumber, it was possible to establish the chemical fingerprint for the functional groups contained by the MO leaf aqueous powder.

##### Identification of phenolic compounds by liquid chromatography-mass spectrometry (LC-MS)

LC-MS experiments were conducted on a 6120 LC-MS analytical system from Ag-ilent (Santa Clara, CA, USA), consisting of 1260 Infinity HPLC (G1322A degasser, G1311B cuaternary pump, G1316A column thermostat, G1365C MWD detector, G7129A au-tosampler Quadrupolar (Q) mass spectrometer, electrospray ionization source (ESI)). LC-MS is connected to a PC computer running the OpenLAB CDS ChemStation Work-station software. The sample solutions were homogenized with a WisdVM-10 vortex mixer (WitegLabortechnik, Germany) and centrifuged for 2 min at 10000 rpm in a ThermoMicro CL17 microcentrifuge (Thermo Fisher Scientific, MA, USA). The supernatant was collected and submitted to LC-MS analysis. All polyphenols were separated on a reverse phase Zorbax Eclipse Plus C18 column (3.0 × 100 mm × 3.5 microns) by gradient elution with a mobile phase consisting of a mixture of 0.1% acetic acid and methanol as follows: 5% methanol up to 5 min, 42% methanol for 38 min, the same proportion up to 41 min and 5% methanol for 1 min as described before [16]. The LC-MS parameters were the following: injection volume, 10 µl; flow rate, 1 mL/min; column temperature, 40 °C; UV detection at 330 and 370 nm; MS detection by electrospray ionization (ESI) in the single ion monitoring mode (SIM); capillary voltage, 3500 V; dry gas flow, 12 L/min at 350 °C; nebulizer pressure, 55 psig; fragmentor 70. Mass spectra were recorded in the negative ion mode. For the quantification of polyphenolic compounds, calibration curves by the external standard method for each compound were conducted. The m/z scale of the mass spectrum was calibrated by use of an external calibration standard ESI Tuning Mix from Agilent (Santa Clara, CA, USA).

#### 4.3.3. Cell Viability Assessment by Alamar Blue Assay

To verify the impact of aqueous extracts of MO leaves on the cells’ viability, the following protocol was applied: i) a number of 1 × 10^4^ cells (HaCaT/A375)/well/200 µL culture media was seeded in 96-well plates and left over night to adhere to the plate; ii) the cells were stimulated for 24 h with five different concentrations of the aqueous extract of MO leaves (20, 100, 250, 500 and 1000 µg/mL) solubilized in distilled water; iii) after the stimulation period, 24 h, the cells were incubated with the Alamar Blue reagent for 3 h; and iv) the absorbance was measured spectrophotometrically (570 and 600 nm) using a microplate reader (xMark™ Microplate Absorbance Spectrophotometer, Biorad, CA, USA). The percentages of viable cells were calculated according to a previous published formula [72,73].

#### 4.3.4. Chorioallantoic Membrane Assay

The antiangiogenic potential of the aqueous extract of MO leaves was evaluated by using the chick embryo chorioallantoic membrane (CAM) assay. The protocol was applied as previously described [74] with some modifications [66,75]. After a thorough cleaning with 70% ethanol of fertilized hen eggs (*Gallus gallus domesticus*), they were incubated at 37 °C, under controlled humidity (50%). On the third day of incubation (also called embryonic day of development, EDD), a small opening was made at one end of the eggs and 3–4 mL of albumen was aspired, thus facilitating the separation of the chorioallantoic membrane. On EDD 4, a window was cut on the upper side of the egg for ease of the experimental procedures. The aqueous extracts of MO leaves in several concentrations (10, 50, 100, 500 and 1000 µg/mL) in distilled water were applied onto the CAM inside previously positioned plastic rings (3 mm in diameter). As a blank sample, the same solvent as for the dilution of the extract was considered. A volume of 10 µL of each solution was applied and stereomicroscopic (Discovery 8 Stereomicroscope, Zeiss, Götingen, Germany) evaluation was assessed. Significant images were registered using the Axio CAM 105 color (Axio Vision SE64. Rel. 4.9.1 Software, Zeiss digital camera (Götingen, Germany)) processed by Zeiss ZEN software, ImageJ (ImageJ Version 1.50e, https://imagej.nih.gov/ij/index.html) and GIMP software (GIMP v 2.8, https://www.gimp.org/). Six groups of samples were tested: (1) distilled water, as positive control, and (2) five concentrations of MO extract (10, 50, 100, 500 and 1000 µg/mL). For each sample, five eggs were used, and all samples were tested in triplicate.

#### 4.3.5. Skin Biophysical Parameters Assessment

In order to evaluate the skin biophysical parameters, all the measurements were carried out on female SKH-1 hairless mice skin, using a Multiprobe Adapter System (MPA5) from Courage-Khazaka (Koln, Germany). The measurements of melanin and erythema levels were obtained using the professional Mexameter^®^ MX 18 probe, while the transepidermal water loss (TWL) and skin hydration were measured using the world-wide popular probes Tewameter^®^ TM 300 and Corneometer^®^ CM 825, respectively, according to a detailed description of the protocol presented in the literature [76]. All the evaluations were performed in triplicate for 30 min by the same operator. The aqueous extract of MO leaves was applied twice a day for two weeks on the mice dorsal skin; there were differences (Δ skin biophysical parameters) between an instant measure and an initial one for every mouse. These different skin biophysical parameters were expressed as mean values and range intervals and they were considered to compare intra- and inter-groups differences.

#### 4.3.6. Statistical Analysis

The statistical programs and software applied in the present study were GraphPad Prism 6 and Origin 2020b (Origin Lab—Data analysis and Graphing Software, Szeged, Ungary). The results were expressed as mean ± standard deviation. For Alamar blue assay, one-way ANOVA analysis was applied to determine the statistical differences, followed by Tukey’s post-test (**** *p* < 0.0001), and for the skin parameters evaluation, a two-way ANOVA analysis was used, followed by a Bonferroni post-test (* *p* < 0.05; ** *p* < 0.001; *** *p* < 0.001).

## 5. Conclusions

The present study reports knowledge about the aqueous extract of *Melissa officinalis* leaves in terms of chemical composition, antioxidant capacity (DPPH) and several biological effects noticed in vitro on human melanoma cells, in ovo on angiogenesis and in vivo at skin level. The in vitro observations indicated a selective cytotoxic potential of the aqueous extract of MO leaves on A375 cells only at the highest concentration tested 1000 μg/mL and no toxic behavior on HaCaT normal cells. The in ovo test pointed out the promising antiangiogenic effects of the aqueous extract of MO leaves by reducing the angiogenic process even at lower concentrations, with no signs of toxicity, thus indicating that MO leaves represent a natural, easily available source for antitumor and antimetastatic agents. The reduction of transepidermal water loss (TWL) and erythema values together with an augmentation of skin hydration make the *Melissa officinalis* aqueous extract a promising candidate that could be used in specific topical formulations for skin disorders characterized by an impairment of these biophysical skin parameters.

## Figures and Tables

**Table 1:** Peak values and functional groups of the aqueous extract of dried MO leaves.

Characteristic Absorptions [cm^−1^]	Functional Group	Bond
3431	Alcohol	O-H stretching vibration (hydroxyl groups H-bonded)
2974	Acid	O-H stretching vibration
1636	Alkene Carbonyl Amide	C=C stretching vibration C=O stretching vibration C=O stretching vibration
1400	Alkane	-C-H bending vibration
1269	Acid	C-O stretching vibration
1076	Ester Cyclic alcohols	C-O stretching vibration C-O stretching vibration
617	Aromatic	=C-H stretching vibration

**Table 2:** Polyphenolic content of an aqueous extract of MO leaves by LC-MS.

Compound Name	Rt (min)	[M − H^+^]^+^ (*m/z*)	MO Extract (ng/mg d.w.)
gentisic acid	2.67	153	NQ
*p*-coumaric acid	10.56	163	NQ
apigenin	36.91	269	38.72
chlorogenic acid	6.45	353	0.31
caffeic acid	6.97	179	0.18
rutin	23.01	609	4.06
ferulic acid	13.91	193	1.25

NQ; Not Quantified.

## Data Availability

Not applicable.

## Sample Availability

Samples of the compounds are not available from authors.
